# The relationship between paediatric nurses' quality of life and intent to leave: The moderating role of hospital type

**DOI:** 10.1002/nop2.1116

**Published:** 2021-11-02

**Authors:** Haitham Khatatbeh, Miklós Zrínyi, András Oláh, Annamária Pakai

**Affiliations:** ^1^ Faculty of Health Sciences Doctoral School of Health Sciences University of Pécs Pécs Hungary; ^2^ Faculty of Health Sciences Basic Health Sciences and Health Visiting Institute of Nursing Sciences University of Pécs Pécs Hungary

**Keywords:** intent to leave, nurse, quality of life, type of hospital

## Abstract

**Background:**

Different types of hospitals exist in Jordan, and each type has its own leadership style. This might affect the nursing workforce in terms of quality of life and intent to leave.

**Aim:**

This study aimed at (1) assessing the relationship between paediatric nurses' quality of life and intent to leave, (2) examining the moderating effect of the hospital type on this relationship, (3) comparing the quality of life of paediatric nurses working at ministry of health and the University‐Affiliated hospitals and (4) exploring the determinants of nurses' intent to leave.

**Design:**

A cross‐sectional, comparative and correlational design was used in this study.

**Methods:**

A multi‐site sample was selected from two types of hospitals in Jordan; the ministry of health and the University‐Affiliated hospitals. A sample of 225 paediatric nurses responded to the brief quality of life questionnaire by the World Health Organization in addition to a group of sociodemographic and work‐related questions.

**Results and conclusion:**

Nurses' quality of life and intent to leave were negatively correlated. This correlation was moderated by the type of hospital. Nurses' quality of life was significantly different in the two types of hospitals. Both salary and nursing care model predicted paediatric nurses' intent to leave. The managerial style and work environment of the ministry of health hospitals should be benchmarked against the University‐Affiliated hospitals.

## 1 INTRODUCTION

Nursing is one of the most stressful professions that affect nurses' quality of life (QOL) negatively (Kandi & Zeinali, 2017) and increase their intent to leave (ITL) the job (Andresen et al., 2017). QOL is a general term that was defined by the world health organization (WHO) as the perceived evaluation of own life surrounded by a cultural, social and environmental framework (World Health Organization, 1996). So, nurses' QOL reflects their well‐being and affects their productivity. ITL means having or making a plan to quit the job (Al‐Faouri et al., 2020; Andresen et al., 2017).

Nurses' QOL is an important topic of study because it is related to many professional issues and variables, such as job satisfaction (Andresen et al., 2017; Khatatbeh, Al‐Dwaikat, et al., 2021; Makabe et al., 2018), work environment (Dos Santos et al., 2018), ITL (Andresen et al., 2017) and burnout (Garbóczy et al., 2021; Hatamipour et al., 2017; Khatatbeh, Pakai, et al., 2021; Khatatbeh, Pakai, Zrínyi, et al., 2020; Khatatbeh, Khasawneh, et al., 2021). On the other hand, ITL is also crucial because it contributes to nurses' shortage, the global problem (Burmeister et al., 2019) and because it is related to poor nursing care and job satisfaction (Al‐Faouri et al., 2020). With the ongoing outbreak of the novel coronavirus (COVID‐19) and the associated work stressors (Khatatbeh, Khasawneh, et al., 2021), increased attention has been paid to nurses' QOL and ITL (Alrawashdeh et al., 2021).

Previous studies established a negative correlation between nurses' QOL and ITL (Andresen et al., 2017; Burmeister et al., 2019; Khatatbeh, Pakai, Pusztai, et al., 2020; Perry et al., 2017). Another study concluded that nurses' ITL is influenced by the type of healthcare institution (Yamaguchi et al., 2016). Also, another study demonstrated that nurses working in a preferred setting with high satisfaction would show less ITL (Al Sabei et al., 2020).

A previous study reported that around 60% of nurses in Jordan have high ITL (Raddaha et al., 2012). In Jordan, several factors were found to be associated with nurses' ITL, such as salaries, leadership style and professional progress (Alhamwan et al., 2015). Similarly, another study found that leadership style affects nurses' intent to stay in Jordan (Al‐Hamdan et al., 2016). The work environment was also correlated with nurses' intent to stay in Jordan (Al‐Hamdan et al., 2017).

For several reasons, we argue that paediatric nursing is even more stressful than general nursing. For example, paediatric nurses deal with underage patients who are afraid of the hospital environment (Khatatbeh, Pakai, et al., 2021). Also, some of those patients are critically ill which makes paediatric nursing more complicated (Khatatbeh, Pakai, et al., 2021). Furthermore, paediatric nurses need to be caring about the parents who are worried about their children. Nevertheless, the COVID‐19 pandemic even yielded more stress on paediatric nurses (Zheng et al., 2021).

**FIGURE 1:**
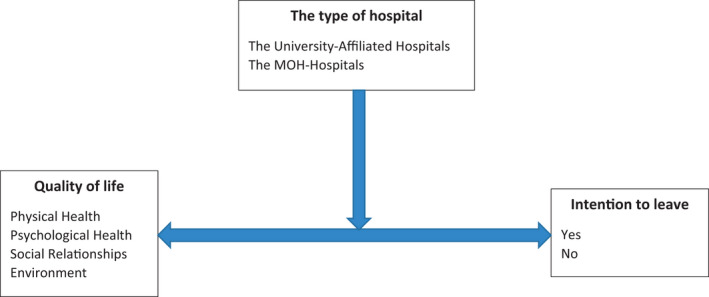
The conceptual model

Different types of hospitals exist in Jordan: the ministry of health (MOH) hospitals, the University‐Affiliated hospitals, the private hospitals and the military hospitals. In Jordan, there are only two University‐Affiliated hospitals. The first one is the *Jordan University Hospital* which belongs to the *University of Jordan*. The second hospital is the *King Abdullah University Hospital* which belongs to the *Jordan University of Science and Technology*. The University‐Affiliated hospitals are independent of other hospitals; they have their management style, salaries, healthcare standards, nursing care model and staffing levels distinct from the MOH hospitals. We argue that the University‐Affiliated hospitals create a more attractive environment that leads to better nurses' QOL and decreases ITL. Based on the relationships established in previous studies (Andresen et al., 2017; Burmeister et al., 2019; Perry et al., 2017; Yamaguchi et al., 2016), we suggest that hospital type might be moderating the relationship between paediatric nurses' QOL and ITL. Figure 1 shows a conceptual model for this proposed relationship.

To the best of our knowledge, no previous studies have explored the moderating role of hospital type in the relationship between paediatric nurses' QOL and ITL, especially paediatric nurses. This study aims to: (1) assess the relationship between paediatric nurses' QOL and ITL, (2) examine the moderating effect of hospital type on the relationship between paediatric nurses' QOL and ITL, (3) compare the quality of life of paediatric nurses working at the MOH and the University‐Affiliated hospitals and (4) explore the determinants of paediatric nurses' intent to leave. So, we hypothesize that paediatric nurses' QOL and ITL are negatively correlated; hospital type moderates the relationship between nurses' QOL and ITL; paediatric nurses working at the University‐Affiliated hospitals have higher scores of QOL than those working at the MOH hospitals; and that there are some determinants in the work environment which contributes to paediatric nurses' ITL.

## 2 METHODOLOGY

### 2.1 Design, setting and sampling

In Jordan, the healthcare system has several sectors: the MOH, University‐Affiliated, military and private sectors. This study covered two types of hospitals in Jordan: the MOH and the University‐Affiliated hospitals. Because 92% of the people in Jordan live in the northern and the central regions (Department of Jordanian Statistics, 2018), seven hospitals in the northern and central regions and one hospital in the southern region were selected to represent the MOH hospitals. Also, one hospital out of the only two University‐Affiliated hospitals in Jordan was selected.

This study used a cross‐sectional, comparative and correlational design to examine the data collected from two types of hospitals in Jordan. An initial pool of 500 paediatric nurses were listed as potential participants. Out of this pool, 300 nurses had randomly been selected, and those meeting inclusion criteria and consenting to participation were approached by the research team via nurse managers in the paediatric units and wards. However, the final sample reflected a more convenient sample because nurses available on the day of data collection were ultimately involved. Finally, 225 paediatric nurses have participated in this study. One hundred and fifty‐eight nurses were working at the MOH hospitals and 67 were working at the University‐Affiliated hospital. Inclusion criteria were (1) having at least 1 year of professional experience as a paediatric nurse; (2) holding at least a 2‐year diploma in nursing; (3) working at the MOH or the University‐Affiliated hospitals and (4) being a Jordanian citizen. The exclusion criteria included having a professional experience of <1 year or holding less than a 2‐year nursing diploma.

Self‐completed questionnaires were handed over to the head or charge nurses, who distributed them to the paediatric nurses. The answered questionnaires were placed in a sealed envelope and returned to nurse managers. Participating nurses were asked to answer the questionnaires independently and not to discuss responses with other colleagues. To ensure confidential participation, paediatric nurses were advised to use the head nurse's office or the meeting room. To allow the nurses working on rotating shifts, questionnaires were collected back on the next day. Data were collected between December 2019–March 2020. A pilot study was conducted on 35 paediatric nurses to assess the feasibility; however, no issues were confronted.

### 2.2 Calculating sample size

A post hoc test was done using G*Power software to ensure adequate statistical power (G*Power, 2020). The *t test* (two groups) with 225 participants (158 and 67), significance set at 0.05 and medium effect size (0.43) provided a power of 0.90, which is statistically enough to make conclusions.

### 2.3 Instruments

Nurses were asked about a set of sociodemographic and work‐related characteristics, including age, gender (Male or Female), marital status (Single/Divorced or Married), educational level (2‐year college, Bachelor's degree or Master's degree), weekly work hours, professional experience, type of hospital (the MOH or University‐affiliated), number of patients they are assigned for, break time per shift, monthly salary perception (Enough or Not enough), time available for family (Enough, Not Enough), nursing care model (Total, Functional, Team, or Other) and exposure to violence (None, Verbal, Emotional, or Physical).

The nursing care model is the way of providing and organizing the nursing care services to the patients (Anneli Pitkanen, 2013; Fernandez et al., 2012). Exposure to violence is any speech, communication or behaviour threatening the paediatric nurses on their duties (Kowalenko et al., 2005).

The brief version of WHO questionnaire (WHOQOL‐BREF) was used to assess the nurses' QOL (World Health Organization, 1996). Using WHOQOL‐BREF, four domains of QOL were assessed: physical health, psychological health, social relationships and environment.

WHOQOL‐BREF is composed of 26 Likert‐type items asking, “how much”, “how satisfied” or “how good” felt during the past 2 weeks. Each item has a possible score ranging from 1–5. The higher the score, the lower the quality of life except for the three negatively phrased scales (3, 4 and 26) that should be reversed (World Health Organization, 1996). In this study, the raw scores on each subscale were converted to percentages using the WHO manual (World Health Organization, 1996). Finally, the total QOL score was calculated by taking the average of the four converted scores.

The WHOQOL‐BREF domains showed acceptable reliabilities; the Cronbach's alpha for the physical health domain was 0.82, for psychological health domain was 0.81, for social relationships domain was 0.68 and for environment domain was 0.80 (Skevington et al., 2004). In this study, the Cronbach's alphas for the four domains were satisfactory. They were 0.70, 0.70, 0.78 and 0.85 for the four domains. Our values were similar to the previous study's Cronbach's alphas, 0.82, 0.81, 0.68 and 0.80 respectively.

Last, ITL was roughly assessed using a single item. Using a dichotomous item, nurses were directly asked if they have any plans to leave the nursing job (yes, no).

### 2.4 Ethical considerations

Before the data collection started, the necessary approvals have been obtained from the central institutional review boards at the MOH and the University‐Affiliated hospital. Consent was requested from each participating nurse.

### 2.5 Data analysis

The Statistical Package for the Social Sciences (SPSS) software (version 20.0) was used to analyse this study's data. The basic descriptive and frequency tests were used to describe the demographic and work‐related characteristics (age, gender, marital status, educational level, weekly work hours, professional experience and type of hospital). Bivariate correlation (Spearman) was used to examine the correlation between the studied variables. Additionally, while controlling the hospital type, the partial correlation test was used for comparison with the result of basic correlation. To compare the environment of the MOH and the University‐Affiliated hospitals, the *t test* was used to compare nurses' QOL. Also, the *binary logistic regression* was used to determine if the studied work‐related variables (number of patients assigned for, weekly work hours, break time per shift, nursing care model, monthly salary perception, time available for family and exposure to violence) predict the ITL.

## 3 RESULTS

### 3.1 Normality of the data

While the Kolmogorov–Smirnov test was significant for QOL, the Shapiro–Wilk test was not significant. This finding, in addition to the histogram shape, means that the QOL variable is normally distributed (Steinskog et al., 2007). However, Kolmogorov–Smirnov and Shapiro–Wilk tests were significant for the other variables (intent to leave, number of patients assigned for, weekly work hours, break time per shift, nursing care model, monthly salary perception, time available for family and exposure to violence), which means they are not normally distributed (Steinskog et al., 2007).

### 3.2 Participants demographics

The sample (*n* = 225) involved 158 nurses from the MOH hospitals and 67 nurses from the University‐Affiliated hospital (see Table 1). The results showed that most participants are females (94.2%) and married (82.7%). The mean age of the participants was 33.6 years, and the mean professional experience was 11.1 years. Regarding their educational level, most of them hold a bachelor's degree (87.6%). Results showed that most of the participants think they do not have enough time for their families (81.8%), and they have been exposed to at least one type of violence: verbal, emotional or physical (74.2%). It was also found that paediatric nurses are assigned for 8.3 patients, on average.

**TABLE 1:** Participants' characteristics and descriptive statistics

Variable	*N*	Percentage (%)
Gender		
Male	11	4.9
Female	212	94.2
Missing	2	0.9
Marital status		
Single/Divorced	39	17.3
Married	186	82.7
Education		
2‐year college	5	2.2
Bachelor's degree	197	87.6
Master's degree	23	10.2
Hospital		
MOH	158	70.2
University‐affiliated	67	29.8
Intent to leave		
Yes	108	48.2
No	116	51.8
Time available for family		
Enough	41	18.2
Not enough	184	81.8
Exposure to violence		
Yes	167	74.2
No	58	25.8
Nursing care model		
Total	154	68.4
Functional	30	13.3
Team	34	15.1
Other	3	1.3
Missing	4	1.8

	M	*SD*
Age (years)	33.6	6.5
Weekly work hours	41.9	5.4
Break time per shift (minute)	34.1	19.7
Professional experience (years)	11.1	6.74
Number of patients assigned for	8.3	17.4
Overall QOL score	45.0	14.2
Physical health domain	43.0	14.0
Psychological health domain	47.7	16.1
Social relationships domain	45.1	20.6
Environment domain	44.4	16.0

### 3.3 Descriptive statistics

The results showed that the paediatric nurses' QOL scores are relatively low. As shown in Table 1, the mean score for the overall QOL was 45.0 (*SD* = 14.2). Regarding the dimensions of QOL, participants scored 43.0 (*SD* = 14.0) on the physical health domain, 47.7 (*SD* = 16.1) on the psychological health domain, 45.1 (*SD* = 20.6) on the social relationships' domain and 44.4 (*SD* = 16.0) on the environment domain.

### 3.4 Correlations

As the data were non‐normally distributed, the Spearman correlation was used and showed some significant correlations between the studied variables. Most importantly, results demonstrated that ITL and QOL scores are significantly and negatively correlated (*r* = −.227, *p* < .01). Another significant correlation was also found between QOL scores and type of hospital (*r* = −.204, *p* < .01).

Controlling the type of hospital, the correlation was also significant between intent to leave and QOL (*r* = −.208, *p* = .001); however, the initial correlation changed from −0.227 to −0.208 showing a moderating effect of hospital type (Table 2).

**TABLE 2:** Bivariate correlation (Spearman)

	(1)	(2)	(3)	(4)	(5)	(6)	(7)	(8)	(9)	(10)
Age (1)	1.000									
Gender (2)	−.089	1.000								
Marital status (3)	.260**	−.022	1.000							
Level of education (4)	−.170**	.048	−.284**	1.000						
Type of hospital (5)	.400**	−.014	.086	−.187**	1.000					
Common work shift (6)	−.567**	−.005	−.178**	.082	−.231**	1.000				
Weekly work hours (7)	.145*	−.046	−.013	.015	.146*	−.255**	1.000			
Professional experience (8)	.906**	−.040	.226**	−.115*	.389**	−.553**	.198**	1.000		
Intention to leave (9)	−.127*	.096	−.057	.109	.084	−.031	−.008	−.102	1.000	
Average QOL (10)	−.012	−.215**	−.078	.066	−.204**	−.031	.039	−.042	−.227**	1.000

*p < .05, **p < .01.

### 3.5 T test

The independent‐samples *t* test showed that the paediatric nurses' QOL score is statistically different between the MOH and the University‐Affiliated hospitals (*t* = 2.81*, p* = .005). In university‐affiliated, the mean QOL score was 49.0 (*SD* = 11.6) compared to 43.2 (*SD* = 14.9) in the MOH hospitals.

### 3.6 The binary logistic regression

In order to find the significant variables predicting paediatric nurses' ITL, binary logistic regression was done. Seven work‐related variables were loaded into the model predicting ITL and the Hosmer and Lemeshow goodness‐of‐fit test was: *X* ^2^ = 18.55, *p* = .017. Within the significant model predicting ITL, only two variables (the monthly salary perception and the nursing care model) were significantly predicting paediatric nurses' ITL. Exposure to violence, the time available for family, number of patients assigned for, weekly work hours and break time per shift were not significant predictors of ITL (Table 3).

**TABLE 3:** The binary logistic regression – Intent to leave (dependent variable), salary, nursing model, exposure to violence, family time, number of patients, working hours and break time (covariates)

Hosmer and Lemeshow goodness‐of‐fit test: *X^2^* = 18.55, *p* = .017					
Covariates	*β*	Standard error	Wald	Significance	Odds ratio
Monthly salary perception	−1.065	.381	7.804	.005	0.345
Nursing care model	−.468	.187	6.276	.012	0.626
Exposure to violence	−.004	.003	2.064	.151	0.996
Time available for family	−.434	.426	1.038	.308	0.648
Number of patients assigned for	.006	.011	0.360	.548	1.006
Weekly work hours	.018	.029	0.379	.538	1.018
Break time per shift	−.005	.008	0.401	.526	0.995

## 4 DISCUSSION

This study aimed to assess the relationship between paediatric nurses' QOL and ITL. The results of this study support the negative correlation between QOL and their ITL nursing jobs. Nurses with better QOL will show less ITL their nursing jobs and vice versa. This finding matches a previous study, which found that a better mental QOL decreases nurses' ITL (Perry et al., 2017). This study is also congruent with a Norwegian study which found that ITL is associated with poor satisfaction about nursing work‐life (Andresen et al., 2017).

According to the hypothesized conceptual model, the second aim of this study was to examine the moderating effect of hospital type on the relationship between nurses' QOL and ITL. The results showed a moderating effect of hospital type on the relationship between nurses' QOL and ITL. This finding is supported by a previous study that established a relationship between the type of healthcare institution and nurses' ITL (Yamaguchi et al., 2016). The moderating effect of hospital type on the relationship between nurses' QOL and ITL can be explained by the distinct strategies and policies for each type of hospital (Al Sabei et al., 2020). The diverse strategies and policies will definitely alter the work environment (Al Sabei et al., 2020; Dos Santos et al., 2018) and affect the working QOL for all employees, including paediatric nurses. Subsequently, QOL will finally affect nurses' satisfaction and ITL (Andresen et al., 2017). Also, the different management style across the different hospitals encompasses various levels of nurses' empowerment that finally affect their satisfaction and ITL (Yamaguchi et al., 2016).

The third aim of this study was to compare QOL of paediatric nurses' working at the MOH and the University‐Affiliated hospitals. The results also showed that paediatric nurses' QOL scores were significantly higher in the University‐Affiliated hospital than in the MOH hospitals. This finding matches what was found in a previous study that the type of hospital and nurses' QOL are correlated (Moradi et al., 2014). On the other hand, this result might conflict with a previous study, which found that nurses working at the University‐Affiliated hospitals have higher stress than those working at the MOH hospitals (Amarneh, 2017). The reason behind the higher stress in the University‐Affiliated hospitals in the study of Amarneh (2017) can be explained by the stricter policies applied in the University‐Affiliated hospitals compared to the MOH hospitals. This finding is supported by another study that found a significant association between nurses' QOL and work environment, such as autonomy and organizational support (Dos Santos et al., 2018).

The last aim of this study was to explore the determinants of paediatric nurses' ITL. Those variables which significantly predicted paediatric nurses' ITL were the monthly salary perception and the nursing care model. Paediatric nurses who think they are getting enough salaries will not have ITL. This finding was congruent with a previous study that found that salary affects nurses' ITL (Alhamwan et al., 2015). Also, the total nursing care model was preferred over the team or functional nursing model. Paediatric nurses in Jordan prefer total patient care over working in teams. Together, these two variables and other variables, which need to be further studied, contribute to the nurses' work environment and make them either want to leave or stay. This finding in addition to the different QOL at the MOH and the University‐Affiliated hospitals indicates that the nurses' work environment might be different in the two types of hospitals in Jordan. That is, the varying work environment and policies might explain the significant differences found in paediatric nurses' QOL between the MOH and the University‐Affiliated hospitals.

## 5 CONCLUSIONS AND IMPLICATIONS

The results showed that paediatric nurses' QOL is negatively correlated with ITL. Also, the hospital type, the MOH or the University‐Affiliated hospitals, moderates the relationship between paediatric nurses' QOL and ITL. Furthermore, paediatric nurses working at the University‐Affiliated hospitals have better QOL than those working at the MOH hospitals. In response to the global nursing shortage and turnover, nurses' QOL improvement should be the target of the healthcare decision‐makers. The managerial style and work environment of the MOH hospitals should be benchmarked against the University‐Affiliated hospitals.

We recommend adopting the University‐Affiliated hospitals' management style in governmental hospitals to improve paediatric nurses' QOL and decrease their ITL. Adopting the University‐Affiliated hospitals' work environment in the MOH hospitals can improve the nurses' QOL and decrease their ITL. This would help in resolving the global nursing shortage (Burmeister et al., 2019) by improving nurses' QOL and reducing ITL. Nurse managers at the MOH hospitals are advised to adopt the nursing care model, the total patient care model, applied usually at the University‐Affiliated hospitals. Salaries of paediatric nurses working at the MOH hospitals need to be reassessed and equalized with salaries of the University‐Affiliated hospitals. To improve paediatric nurses' QOL, nursing executives should work on improving physical health, psychological health, social relationships and work environment. The possible interventions might include applying vertical rotation by promoting the nurse to a higher hierarchical position. Another intervention is the horizontal rotation by assigning nurses to a new unit/ward. Quality circle is a voluntary managerial technique, in which the staff participates with their manager in a brainstorming activity to discover and solve work‐related problems (Christman, 2003).

Future research should test the suggested interventions to improve paediatric nurses' QOL and decrease ITL. The interventions include vertical rotation, horizontal rotation and quality circles.

### 5.1 Limitations

The convenient sampling technique and cross‐sectional design used might limit the results' generalization. Since this study is a part of a larger study about burnout and quality of life, ITL assessed using a single scale is an additional limitation. Also, the self‐reported nature of the analysed data is another limitation. Last, the study has been done in a specific socio‐economic context which might not be generalizable to other contexts. However, the multi‐site sample is a privilege.

## CONFLICT OF INTEREST

The authors declare that they have no conflict of interest.

## AUTHOR CONTRIBUTIONS

All authors are responsible for the reported research and have approved the manuscript as submitted. **Haitham Khatatbeh:** Conceptualization, Data collection, Data curation, Data analysis and Writing Original draft preparation; **Miklós Zrínyi:** Conceptualization, Methodology, Data curation, Data analysis, Writing Original draft preparation and Supervision and **Annamária Pakai and András Oláh:** Conceptualization, Writing reviewing and editing and Supervision.

## ETHICAL APPROVAL

Ethical approval was obtained before research implementation both from the Scientific Research Committee of the Jordanian Ministry of Health (reg. # 21114) and from the Ethics Committee of King Abdullah University Hospital (reg. # 13–3–17).

## Data Availability

The raw data that supports the results of this research are available from the corresponding author upon a reasonable request.
